# Circulating choline pathway nutrients and risk of moyamoya disease

**DOI:** 10.3389/fnut.2022.953426

**Published:** 2022-08-01

**Authors:** Peicong Ge, Yaobo Zhao, Yuanren Zhai, Qian Zhang, Xun Ye, Jia Wang, Rong Wang, Yan Zhang, Dong Zhang, Jizong Zhao

**Affiliations:** ^1^Department of Neurosurgery, Beijing Tiantan Hospital, Capital Medical University, Beijing, China; ^2^China National Clinical Research Center for Neurological Diseases, Beijing, China; ^3^Center of Stroke, Beijing Institute for Brain Disorders, Beijing, China; ^4^Beijing Key Laboratory of Translational Medicine for Cerebrovascular Disease, Beijing, China; ^5^Beijing Translational Engineering Center for 3D Printer in Clinical Neuroscience, Beijing, China; ^6^Department of Neurosurgery, Beijing Hospital, Beijing, China; ^7^Savaid Medical School, University of Chinese Academy of Sciences, Beijing, China

**Keywords:** moyamoya, choline, betaine, risk factors, subtypes

## Abstract

**Background:**

Circulating choline pathway nutrients play a critical role in first stroke and recurrent stroke. However, there is limited information available on the effects of choline pathway nutrients on the risk of moyamoya disease (MMD) and its subtypes. We investigated the association between circulating choline and betaine and the incident risk of MMD and its subtypes.

**Methods:**

The case-control study enrolled 385 patients with MMD [i.e., 110 transient ischemic attack (TIA)-type MMD, 157 infarction-type MMD, and 118 hemorrhagic-type MMD] and 89 matched healthy controls.

**Results:**

Serum choline and betaine were inversely related to the risk of MMD and its subtypes. The risk of MMD was decreased with each increment in choline level [per 1 μmol increase: odds ratio (OR), 0.756; 95% CI, 0.678–0.843] and betaine level (per 1 μmol increase: OR, 0.952; 95% CI, 0.932–0.972), respectively. When choline and betaine were assessed as quartiles, compared with the lowest quartile of serum choline and betaine levels, those in the highest quartile had a significantly decreased risk of MMD (choline, Q4 vs. Q1: OR, 0.023; 95% CI, 0.005–0.118; betaine, Q4 vs. Q1: OR, 0.058; 95% CI, 0.018–0.184).

**Conclusions:**

Serum choline and betaine were associated with the decreased risk of MMD and its subtypes.

## Introduction

Moyamoya disease (MMD) is a rare cerebrovascular disorder, which is characterized by progressive stenosis/occlusion of the internal carotid arteries and their proximal branches, with the formation of collateral abnormal vascular network formation at the basal part of the brain (1). Although MMD is a rare cerebrovascular disorder, it is the main cause of stroke in children and adolescents in East Asian populations (2). There are two well-established MMD phenotypes: one is the ischemic type and the other is the hemorrhagic type (3).

The etiology of MMD is currently unknown and immune, inflammation, genetic, and other factors may have all contributed to the development and progression of MMD (2, 4, 5). A strong association has been found between RNF213 p.R4810K variant and MMD. Approximately 90% of Japanese patients, 79% of Korean patients, and 23% of Chinese patients showed this variant (6–8). Obviously, the incidence of p.R4810K variant was much lower in China than in other East Asian countries. Therefore, in addition to genetic factors, there are other factors contributing to MMD in China. In our recent case-control study, we found that several traditional modifiable risk factors, such as albumin (ALB), homocysteine (Hcy), body mass index (BMI), and high-density lipoprotein (HDL), were correlated with the risk of MMD (9). Nevertheless, the traditional risk factors cannot explain all of the MMD risk factors. Identification of novel risk factors is of urgent necessity, particularly modifiable risk factors.

In recent years, choline pathway metabolites have been of extensive interest and play an important role in many physiological and pathological processes of cardiovascular and cerebrovascular diseases (10–12). Choline is an essential nutrient with diverse biological functions, such as cell membrane integrity, cholinergic neurotransmission, lipid transport, and one-carbon metabolism (13). Betaine is a product of an irreversible oxidation reaction of choline, and it participates as a methyl donor in choline metabolism, which increases the remethylation of Hcy to methionine and influences DNA and histone methylation (14). Abnormal choline metabolism was implicated in the development and progression of processes of cardiovascular and cerebrovascular diseases. The current findings raise questions regarding the role of choline pathway metabolites in MMD risk. Therefore, we conducted this prospective study to investigate the association between serum choline and betaine and the risk of MMD and its subtypes.

## Methods

In this study, we recruited prospectively consecutive adult patients with MMD aged 18 years or older at the Department of Neurosurgery, Beijing Tiantan Hospital, Capital Medical University from September 1, 2020 to December 31, 2021. All participants provided written informed consent. The protocol of this study was approved by the Ethics Committee of Beijing Tiantan Hospital, Capital Medical University.

### Study participants

**Figure 1:**
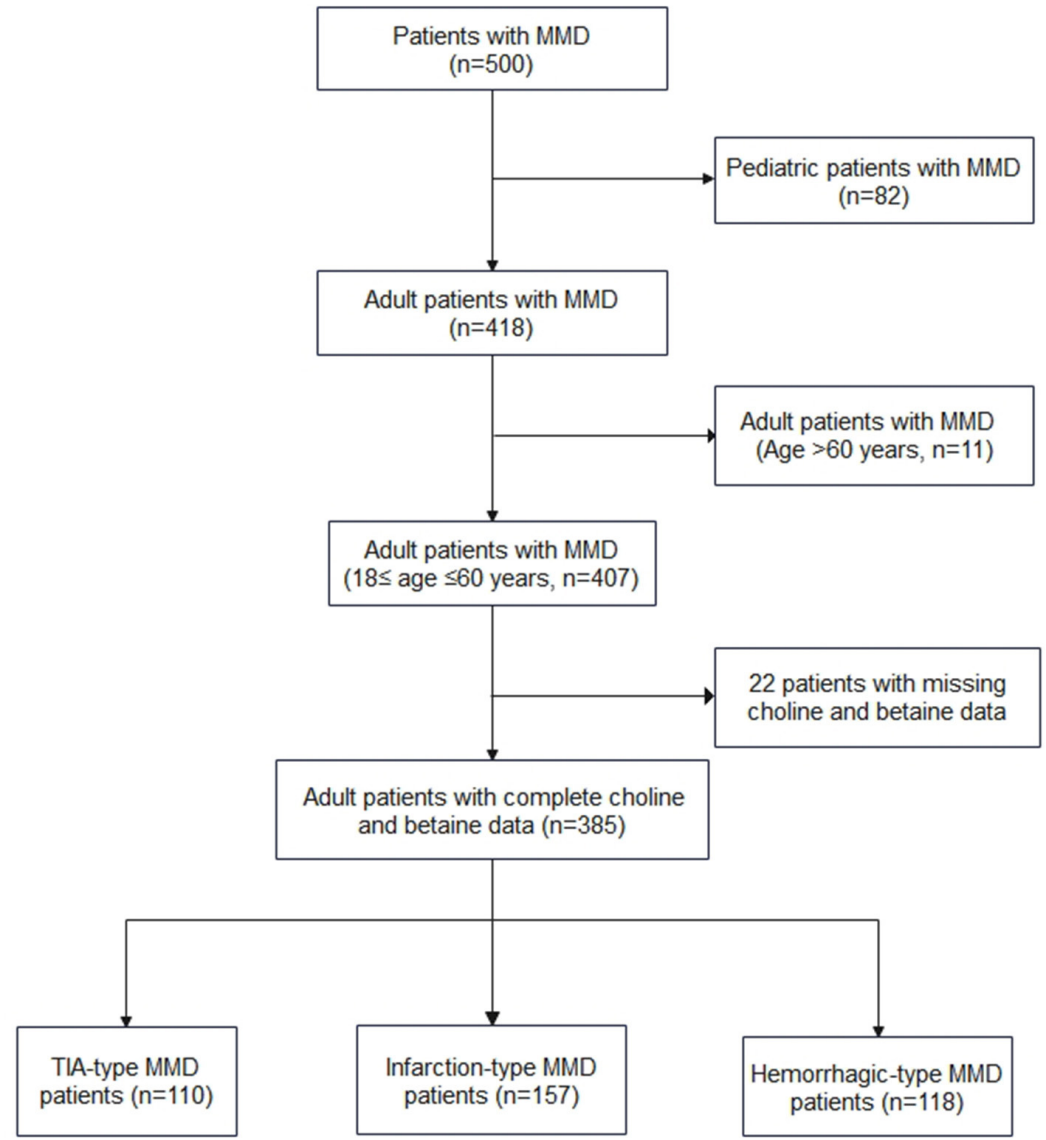
Flow diagram of the study participants.

All patients with MMD were diagnosed using digital subtraction angiography according to the Japanese guidelines published in 2012 (15), and both unilateral and bilateral MMDs were enrolled. From September 1, 2020 to December 31, 2021, 500 patients (that included 418 adult patients) with MMD received their treatment at our center and 385 adult patients with complete choline and betaine measurements were enrolled in the study (Figure 1). Among the 385 adult patients, 110 cases were of transient ischemic attack (TIA)-type MMD, 157 cases were of infarction-type MMD, and 118 cases were of hemorrhagic-type MMD. In the control group, age-matched healthy individuals who came for routine checkups were recruited. Based on interviews with these individuals and their families, none of them had MMD or heart disease.

### Baseline data collection

All subject data were collected by trained research coordinators *via* questionnaires and independent chart reviews, which included age, sex, heart rate, systolic blood pressure (SBP), diastolic blood pressure (DBP), BMI, medical history (hypertension, diabetes, hypercholesterolemia, cigarette smoking, and alcohol consumption). Fasting blood was used to determine white blood cell count (WBC), lymphocyte (LY) count, neutrophil count, platelet count, glucose, ALB, creatinine, uric acid, triglyceride, total cholesterol, HDL cholesterol, low-density lipoprotein (LDL) cholesterol, Hcy, and estimated glomerular filtration rate (eGFR) by using an automatic clinical analyzer. RNF213 p.R4810K variant was detected. The primers were designed as follows: RNF213-4810F (rs112735431): 5′-GCCCTCCATTTCTAGCACAC-3′; and RNF213-4810R: 5′-AGCTGTGGCGAAAGCTTCTA-3′. Within 24 h of patients' admission to the hospital, fasting blood samples were drawn into serum separation tubes and ethylenediaminetetraacetic acid (EDTA) anticoagulation blood collection tubes and then all the blood samples were stored at −80°C in the Central Laboratory of Beijing Tiantan Hospital, Capital Medical University until testing was performed. Liquid chromatography-mass spectrometry was used to measure serum levels of free choline and betaine. Laboratory technicians who measured serum-free choline and betaine were blinded to baseline characteristics.

### Statistical analysis

Baseline characteristics were presented and compared between cases and controls. Continuous variables are expressed as the means with SD. Group comparisons were carried out using Student's *t*-tests, Mann-Whitney U tests, or chi-squared tests, as appropriate. The generalized linear regression analysis was used to test for trends across the choline and betaine for continuous variables, and the Cochran-Armitage trend χ^2^-test or Mantel-Haenszel test was applied for categorical variables, as appropriate. We performed three logistic regression models to identify the independent risk factors of MMD and its subtypes: the crude model was an unadjusted logistic regression model; model 1 was adjusted for age, sex, heart rate, SBP, DBP, and BMI; model 2: model 1 was further adjusted for WBC count, LY count, neutrophil count, platelet count, ALB, creatinine uric acid, triglyceride, total cholesterol, HDL cholesterol, LDL cholesterol, apolipoprotein A (ApoA), apolipoprotein B (ApoB), Hcy, and eGFR. Statistical analysis was performed using SPSS software (version 20.0; IBM Corporation, Armonk, NY, USA) and R version 4.1.2. *p* < 0.05 was considered statistically significant.

## Results

### Study participants and characteristics

This analysis included 385 MMD cases (110 cases of TIA-type MMD, 157 cases of infarction-type MMD, and 118 cases of hemorrhagic-type MMD) and 89 matched controls with complete choline and betaine measurements. A total of 192 (40.5%) men and 282 (59.5%) women were included, and the median age was 41 years [interquartile range (IQR), 33–49 years]. The median plasma choline and betaine concentrations were 11.40 μmol/L (IQR, 8.70–13.30 μmol/L) and 33.70 μmol/L (IQR, 25.50–42.30 μmol/L), respectively.

Baseline population characteristics of MMD and controls are presented in Table 1. Patients with MMD had more risk factors for stroke than the healthy controls. MMD and its subtypes had higher levels of SBP, triglyceride, and ApoA and a higher prevalence of hypertension, diabetes mellitus, hypercholesterolemia, cigarette smoking, alcohol consumption, and evaluated Hcy (*p* < 0.05 for all). In addition, patients with MMD had lower levels of HDL cholesterol, choline, and betaine (*p* < 0.05 for all).

**Table 1:** Clinical and laboratory characteristics in patients with moyamoya disease (MMD) and healthy controls.

**Variables**	**Controls**	**MMD**	***p***	**TIA-type MMD**	***p***	**Infarction-type MMD**	***p***	**Hemorrhagic-type MMD**	***p***
	**(*****n*** = **89)**	**(*****n*** = **385)**		**(*****n*** = **110)**		**(*****n*** = **157)**		**(*****n*** = **118)**	
Age, years	39 (31–50)	42 (34–49)	0.377	42.5 (34–48)	0.718	42 (34–50)	0.258	40.5 (33–50)	0.518
Sex, male	37 (41.6)	155 (40.3)	0.820	43 (39.1)	0.834	73 (46.4)	0.455	39 (33.1)	0.208
RNF213 p.R4810K	0/89	70/343	<0.001^*^	22/100	<0.001^*^	26/141	<0.001^*^	22/102	<0.001^*^
**Clinical features**									
Heart rate, bpm	77 ± 10	79 ± 6	0.100	79 ± 6	0.142	78 ± 6	0.462	79 ± 6	0.030^*^
SBP, mmHg	124 ± 12	132 ± 13	<0.001^*^	133 ± 12	<0.001^*^	134 ± 13	<0.001^*^	129 ± 12	0.001^*^
DBP, mmHg	78 ± 8	82 ± 9	0.001^*^	81 ± 9	0.016^*^	83 ± 10	<0.001^*^	80 ± 9	0.094
Body mass index, kg/m^2^	24.0 ± 3.4	25.5 ± 4.5	0.008^*^	25.8 ± 4.96	0.011^*^	26.0 ± 4.4	<0.001^*^	24.5 ± 4.2	0.638
**Medical history**									
Hypertension	0 (0.0)	137 (35.6)	<0.001^*^	34 (30.9)	<0.001^*^	72 (45.9)	<0.001^*^	31 (26.3)	<0.001^*^
Diabetes mellitus	0 (0.0)	58 (15.1)	<0.001^*^	14 (12.7)	<0.001 ^*^	40 (25.5)	<0.001^*^	4 (3.4)	<0.001^*^
Hypercholesterolemia	0 (0.0)	55 (14.3)	<0.001^*^	16 (14.5)	<0.001^*^	72 (19.1)	<0.001^*^	31 (7.6)	<0.001^*^
Cigarette smoking	2 (2.2)	73 (19.0)	<0.001^*^	19 (17.3)	0.001^*^	37 (23.6)	<0.001^*^	17 (14.4)	<0.001^*^
Alcohol consumption	0 (0.0)	43 (11.2)	<0.001^*^	13 (11.8)	<0.001^*^	22 (14.0)	<0.001^*^	8 (6.8)	<0.001^*^
**Laboratory results**									
WBC count, 10^9^/L	6.09 ± 1.49	7.08 ± 2.01	<0.001^*^	7.12 ± 1.65	<0.001^*^	7.32 ± 2.17	<0.001^*^	6.73 ± 2.04	0.048^*^
Lymphocyte count, 10^9^/L	1.89 ± 0.50	2.00 ± 0.61	0.194	2.16 ± 0.62	0.001^*^	2.02 ± 0.57	0.132	1.83 ± 0.63	0.271
Neutrophil count, 10^9^/L	3.69 ± 1.32	4.49 ± 1.96	<0.001^*^	4.40 ± 2.07	<0.001^*^	4.73 ± 2.00	<0.001^*^	4.42 ± 1.36	<0.001^*^
Platelet count, × 10^9^/L	248 ± 60	256 ± 70	0.261	252 ± 56	0.321	266 ± 79	0.079	246 ± 66	0.935
Glucose, mmol/L	5.08 ± 0.54	5.49± 1.52	0.341	5.50 ± 1.60	0.236	5.80 ± 1.75	0.007^*^	5.05 ± 0.89	0.057
Albumin, g/L	45.36 ± 3.32	45.2 ± 3.0	0.296	45.40 ± 2.91	0.243	45.14 ± 3.08	0.410	45.13 ± 3.02	0.477
Creatinine, μmol/L	58.29 ± 15.10	56.86 ± 13.86	0.110	56.20 ± 13.14	0.090	57.64 ± 13.87	0.489	56.45 ± 14.56	0.289
Uric acid, μmol/L	309.68 ± 74.23	314.1 ± 89.4	0.767	314.48 ± 89.23	0.685	325.75 ± 91.26	0.135	298.28 ± 85.12	0.289
Triglyceride, mmol/L	1.10 ± 0.73	1.43 ± 1.06	<0.001^*^	1.55 ± 1.45	<0.001^*^	1.39 ± 0.87	<0.001^*^	1.36 ± 0.84	0.004^*^
Total cholesterol, mmol/L	4.66 ± 0.72	4.26 ± 0.97	<0.001^*^	4.24 ± 0.97	0.008^*^	4.02 ± 0.93	<0.001^*^	4.60 ± 0.84	0.294
HDL cholesterol, mmol/L	1.52 ± 0.29	1.32 ± 0.29	<0.001^*^	1.32 ± 0.28	<0.001^*^	1.27 ± 0.28	<0.001^*^	1.37 ± 0.29	<0.001^*^
LDL cholesterol, mmol/L	2.73 ± 0.65	2.45 ± 0.83	0.001^*^	2.39 ± 0.81	0.001^*^	2.26 ± 0.79	<0.001^*^	2.77 ± 0.83	0.817
ApoA, g/L	1.40 ± 0.19	1.29 ± 0.26	<0.001^*^	1.32 ± 0.26	0.024^*^	1.28 ± 0.25	<0.001^*^	1.30 ± 0.27	0.001^*^
ApoB, g/L	0.82 ± 0.20	0.84 ± 0.22	0.267	0.82 ± 0.20	0.519	0.82 ± 0.23	0.993	0.90 ± 0.24	0.016^*^
Homocysteine, μmol/L	11.24 ± 6.26	13.31 ± 6.89	0.002^*^	13.13 ± 7.61	0.154	13.74 ± 7.13	<0.001^*^	12.90 ± 5.81	0.007^*^
Elevated homocysteine (≥15.00)	8 (9.0)	93 (21.4)	0.002^*^	27 (24.5)	0.004^*^	40(25.5)	0.002^*^	26(22.0)	0.012^*^
eGFR mL/min per 1.73 m^2^	124.82 ±15.08	125.07 ± 15.22	0.494	125.93 ± 14.42	0.241	125.01 ± 15.53	0.750	124.35 ± 15.62	0.723
Betaine, μmol/L	46.73 ± 37.09	32.15 ± 14.42	<0.001^*^	31.93 ± 15.21	<0.001^*^	32.72 ± 13.90	<0.001^*^	31.58 ± 14.43	<0.001^*^
Choline, μmol/L	12.75 ± 2.33	10.29 ± 3.91	<0.001^*^	10.08 ± 4.01	<0.001^*^	10.57 ± 3.92	<0.001^*^	10.13 ± 3.79	<0.001^*^
Betaine/Choline ratio	3.68 ± 3.64	3.02 (2.51-3.65)	0.104	3.23 ± 1.08	0.302	3.18 ± 1.02	0.152	3.26 ± 1.33	0.092

*Continuous variables are expressed as mean ± SDs. Categorical variables are expressed as n (%)*.

* *p < 0.05*.

**Figure 2:**
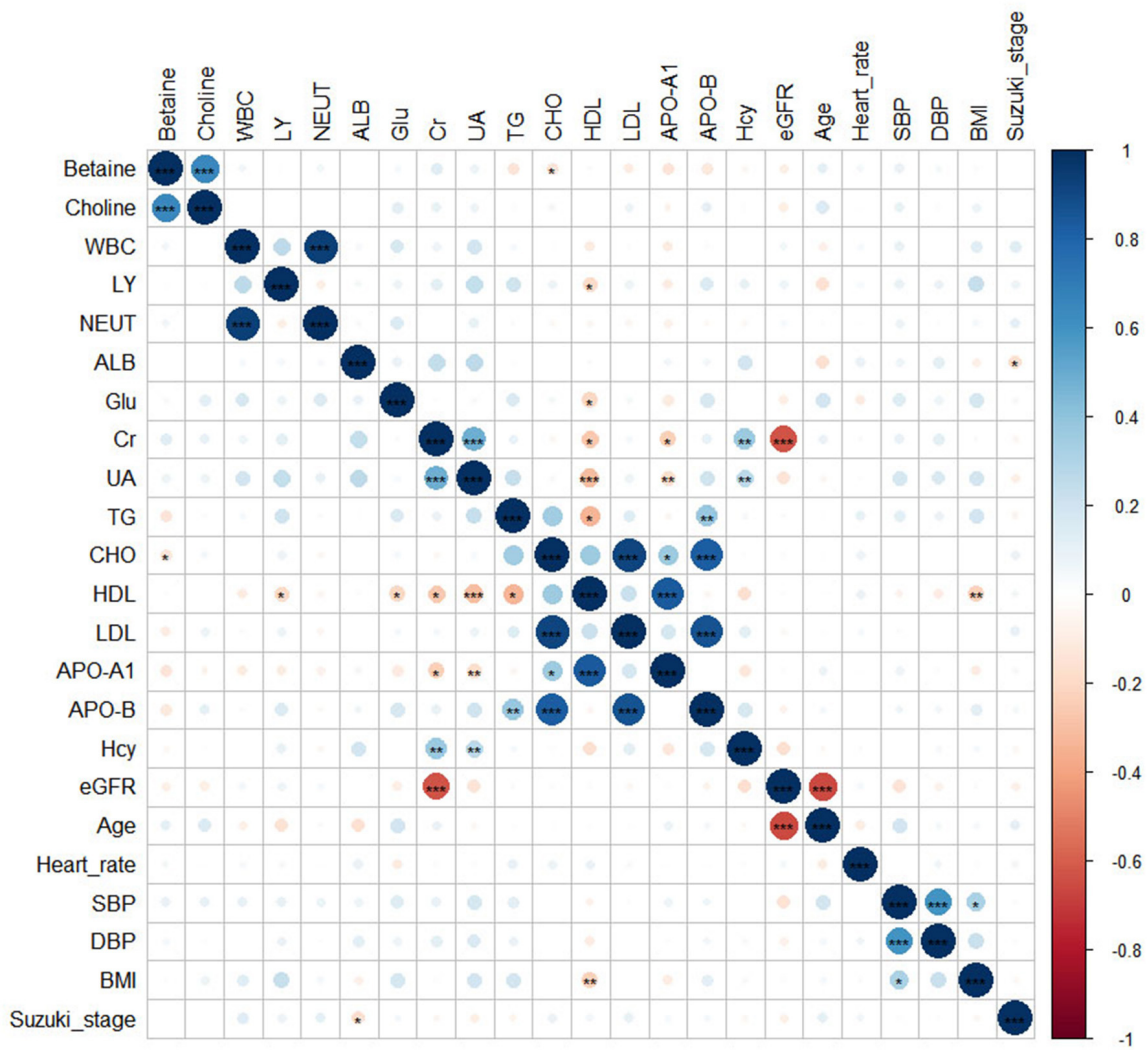
The heatmap showed the correlations between betaine, choline, and other risk factors. Significance: ***p < 0.001; **p < 0.01; *p < 0.05.

Clinical characteristics of the patients with MMD according to the choline and betaine quartiles are shown in Table 2. Patients with higher choline levels tended to be men and older; had higher levels of glucose and betaine. Participants with higher betaine levels tended to be men and older, had higher creatinine and choline levels, and lower levels of triglyceride, total cholesterol, LDL cholesterol, HDL cholesterol, ApoA, ApoB, and eGFR. The correlation between betaine, choline, and other risk factors was done by Pearson's correlation coefficient test. There is a significant positive association between betaine and choline or total cholesterol (Figure 2).

**Table 2:** Baseline characteristics of patients with moyamoya disease (MMD) according to quartiles of serum choline pathway nutrients.

**Characteristics**	**Betaine**					**Choline**				
	**Q1 (4.25–24.10)**	**Q2 (24.10–31.80)**	**Q3 (31.8–40.10)**	**Q4 (40.10–98.70)**	***p*** **trend**	**Q1** **(1.63–8.12)**	**Q2 (8.12–10.80)**	**Q3 (10.80–13.00)**	**Q4 (13.00–22.00)**	***p*** **trend**
Patients	96	97	96	96		96	97	96	96	
RNF213 p.R4810K	22/91	13/83	14/84	21/85	0.939	19/90	22/83	13/85	16/85	0.421
Age, years	38.8 ±10.7	41.7 ± 15.1	41.3 ± 10.4	42.3 ± 10.0	0.029^*^	38.9 ± 10.7	40.3 ± 10.5	42.5 ± 9.6	42.5 ± 9.4	0.005^*^
Sex, male	22 (22.9)	38 (39.2)	45 (46.9)	50 (52.1)	<0.001^*^	31 (32.3)	37 (38.1)	34 (35.4)	53 (55.2)	0.010^*^
**Clinical features**										
Heart rate, bpm	79 ± 6	78 ± 6	78 ± 7	79 ± 6	0.630	79 ± 6	79 ± 7	79 ± 5	78 ± 6	0.882
SBP, mmHg	130 ± 11	132 ± 15	134 ±13	133 ± 12	0.103	131 ± 14	132 ± 13	132 ± 11	134 ± 13	0.110
DBP, mmHg	80 ± 9	83 ± 11	83 ± 9	81 ± 9	0.346	82 ± 10	83 ± 9	81 ± 8	81 ± 10	0.588
Body mass index, kg/m2	24.9 ± 3.9	25.7 ± 5.0	26.1 ± 4.8	25.1 ± 4.3	0.687	25.4 ± 4.6	25.0 ± 4.2	25.0 ± 3.4	26.4 ± 5.6	0.127
**Medical history**										
Hypertension	28 (29.2)	39 (40.2)	32 (33.3)	38 (39.6)	0.329	30 (31.3)	36 (37.1)	39 (40.6)	32 (33.3)	0.696
Diabetes mellitus	11 (11.5)	16 (16.5)	17 (17.7)	14 (14.6)	0.571	10 (10.4)	12 (12.4)	15 (15.6)	21 (21.9)	0.043^*^
Hypercholesterolemia	11 (11.5)	12 (12.4)	18 (18.8)	14 (14.6)	0.387	12 (12.5)	17 (17.5)	12 (12.5)	14 (14.6)	0.948
Cigarette smoking	10 (10.4)	20 (20.6)	23 (24.0)	20 (20.8)	0.091	14 (14.6)	15 (15.5)	14 (14.6)	30 (31.3)	0.016^*^
Alcohol consumption	8 (8.3)	15 (15.5)	12 (12.5)	8 (8.3)	0.855	11 (11.5)	12 (12.4)	14 (14.6)	6 (6.3)	0.413
**Laboratory results**										
WBC count, 109/L	6.88 ± 1.99	6.89 ± 1.92	7.28 ± 2.11	7.26 ± 2.09	0.098	7.15 ± 2.12	7.10 ± 2.07	6.79 ± 1.60	7.27 ± 2.17	0.948
Lymphocyte count, 109/L	1.96 ± 0.57	2.03 ± 0.64	2.01 ± 0.59	2.00 ± 0.65	0.724	2.02 ± 0.64	1.95 ± 0.54	2.00 ± 0.60	2.04 ± 0.67	0.672
Neutrophil count, 109/L	4.39 ± 1.97	4.34 ± 1.75	4.71 ± 2.02	4.72 ± 1.98	0.725	4.57 ± 2.00	4.62 ± 1.88	4.30 ± 1.33	4.68 ± 2.15	0.982
Platelet count, × 109/L	265 ± 68	261 ± 68	235 ± 51	261 ± 85	0.211	250 ± 59	255 ± 58	265 ± 84	252 ± 74	0.581
Glucose, mmol/L	5.28 ± 1.12	5.72 ± 1.89	5.37 ± 1.41	5.57 ± 1.54	0.443	5.38 ± 1.24	5.34 ± 1.24	5.38 ± 1.48	5.84 ± 1.96	0.040^*^
Albumin, g/L	45.1 ± 2.9	45.4 ± 3.0	45.5 ± 3.0	44.8 ± 3.0	0.621	45.4 ± 2.8	45.3 ± 3.1	44.9 ± 3.3	45.2 ± 2.8	0.585
Creatinine, μmol/L	54.12 ± 14.44	54.94 ± 13.20	58.74 ± 12.57	59.67 ± 14.54	0.001^*^	55.66 ± 14.14	56.41 ± 12.95	56.31 ± 13.77	59.07 ± 14.5	0.110
Uric acid, μmol/L	303.16 ± 84.9	309.35 ± 96.9	317.6 ± 89.0	326.3 ± 85.8	0.057	305.7 ± 88.8	314.7 ± 95.3	306.9 ± 86.1	329.2 ± 86.3	0.125
Triglyceride, mmol/L	1.54 ± 1.16	1.60 ± 1.39	1.34 ± 0.71	1.24 ± 0.81	0.015^*^	1.44 ± 1.10	1.46 ± 1.36	1.41 ± 0.93	1.41 ± 0.78	0.781
Total cholesterol, mmol/L	4.44 ± 0.96	4.39 ± 0.97	4.21 ± 0.98	4.00 ± 0.93	0.001^*^	4.19 ± 0.88	4.22 ± 1.08	4.19 ± 0.98	4.44 ± 0.92	0.104
HDL cholesterol, mmol/L	1.35 ± 0.33	1.31 ± 0.27	1.31 ± 0.29	1.19 ± 0.25	0.197	1.30 ± 0.27	1.34 ± 0.34	1.31 ± 0.27	1.32 ± 0.27	0.818
LDL cholesterol, mmol/L	2.58 ± 0.85	2.53 ± 0.80	2.43 ± 0.84	2.26 ± 0.82	0.007^*^	2.40 ± 0.79	2.41 ± 0.89	2.38 ± 0.86	2.62 ± 0.78	0.103
ApoA, g/L	1.35 ± 0.29	1.31 ± 0.24	1.28 ± 0.26	1.24 ± 0.24	0.001^*^	1.30 ± 0.25	1.32 ± 0.28	1.28 ± 0.23	1.28 ± 0.28	0.375
ApoB, g/L	0.88 ± 0.24	0.87 ± 0.20	0.83 ± 0.23	0.79 ± 0.22	0.003^*^	0.82 ± 0.21	0.83 ± 0.24	0.83 ± 0.22	0.88 ± 0.23	0.074
Homocysteine, μmol/L	13.19 ± 6.22	13.69 ± 8.39	13.37 ± 6.37	12.97 ± 6.42	0.759	13.20 ± 5.62	14.30 ±8.62	12.40 ± 5.72	13.32 ± 7.13	0.618
HHcy (≥15.00)	24 (25.0)	23 (23.7)	24 (25.0)	22 (22.9)	0.824	24 (25.0)	33 (34.0)	17 (17.7)	19 (19.8)	0.151
eGFR mL/min per 1.73 m^2^	127.44 ± 17.85	125.74 ± 14.38	124.04 ± 13.87	123.05 ± 14.33	0.033^*^	127.20 ± 17.59	125.69 ± 14.21	123.35 ± 15.02	124.04 ± 13.73	0.089
Betaine	14.31 ± 6.37	27.99 ± 2.20	36.15 ± 2.48	50.18 ± 10.25	<0.001^*^	17.80 ± 11.39	33.74 ± 11.67	36.73 ± 12.58	40.30 ± 10.76	<0.001^*^
Choline	5.89 ± 3.48	10.71 ± 2.65	11.90 ± 2.33	12.67 ± 3.02	<0.001^*^	4.96 ± 2.25	9.50 ± 0.78	11.86 ± 0.55	14.86 ± 1.71	<0.001^*^
**Clinical manifestation**										
Ischemic type	65 (67.7)	62 (63.9)	71 (74.0)	69 (71.9)	0.347	66 (68.8)	65 (67.0)	67 (69.8)	69 (71.9)	0.612
Infarction	39 (40.6)	37 (38.1)	39 (40.6)	42 (43.8)	0.643	37 (38.5)	42 (43.3)	36 (37.5)	42 (43.8)	0.702
TIA	26 (27.1)	25 (25.8)	32 (33.3)	27 (28.1)	0.649	29 (30.2)	23 (23.7)	31 (32.3)	27 (28.1)	0.919
Hemorrhagic type	31 (32.3)	35 (36.1)	25 (26.0)	27 (298.1)	0.347	30 (31.3)	32 (33.0)	29 (30.2)	27 (28.1)	0.612
IVH	16 (16.7)	16 (16.5)	11 (11.5)	13 (13.5)	0.432	14 (14.6)	14 (14.4)	16 (16.7)	12 (12.5)	0.827
ICH+IVH	9 (9.4)	8 (8.2)	4 (4.2)	8 (8.3)	0.600	8 (8.3)	10 (10.3)	6 (6.3)	5 (5.2)	0.328
ICH	6 (6.3)	8 (8.2)	9 (9.4)	6 (6.3)	0.935	8 (8.3)	5 (5.2)	6 (6.3)	10 (10.4)	0.592
SAH	0 (0.0)	3 (3.1)	1 (1.0)	0 (0.0)	0.695	0 (0.0)	3 (3.1)	1 (1.0)	0 (0.0)	0.695
mRS score <2 at admission	38 (39.6)	51 (52.6)	48 (50.0)	35 (36.5)	0.642	36 (37.5)	43 (44.3)	50 (52.1)	43 (44.8)	0.253
Unilateral lesions	10 (10.4)	11 (11.3)	11 (11.5)	16 (16.7)	0.273	9 (9.4)	10 (10.3)	12 (12.5)	17 (17.7)	0.114
Suzuki stage^†^					0.993					0.372
0–2	77 (40.1)	91 (46.9)	78 (40.6)	88 (45.8)		81 (42.2)	69 (35.6)	95 (49.5)	89 (46.4)	
3–4	89 (46.4)	87 (44.8)	92 (47.9)	73 (38.0)		91 (47.4)	99 (51.0)	73 (38.0)	78 (40.6)	
5–6	26 (13.5)	16 (16.8)	22 (23.2)	31 (32.6)		20 (10.4)	26 (13.4)	24 (12.5)	25 (13.0)	

* *p < 0.05*.

† *770 hemispheres in 385 patients with MMD*.

### Choline pathway metabolites and risk of MMD

The associations of serum choline and betaine with the risks of MMD are presented in Tables 3, 4. Both serum choline and betaine were inversely associated with the risk of MMD. Overall, after fully adjusting for age, sex, heart rate, SBP, DBP, BMI, WBC count, LY count, neutrophil count, platelet count, ALB, creatinine uric acid, triglyceride, total cholesterol, HDL cholesterol, LDL cholesterol, ApoA, ApoB, Hcy, and eGFR, the risk of MMD was decreased with each increment in choline level [per 1 μmol increase: odds ratio (OR), 0.756; 95% CI, 0.678–0.843] and betaine level (per 1 μmol increase: OR, 0.952; 95% CI, 0.932–0.972), respectively. When choline and betaine were assessed as quartiles, compared with the lowest quartile of serum choline and betaine levels, those in the highest quartile had a significantly decreased risk of MMD (choline, Q4 vs. Q1: OR, 0.023; 95% CI, 0.005–0.118; betaine, Q4 vs. Q1: OR, 0.058; 95% CI, 0.018–0.184).

**Table 3:** The association between baseline choline levels and the risk of moyamoya disease (MMD) and its subtypes.

**Choline**, μ**mol/L**	**No. of events (%)**	**Crude model**		**Model 1**		**Model 2**	
		**OR (95%CI)**	***P***	**OR (95%CI)**	***p***	**OR (95%CI)**	***p***
**MMD overall**							
**Continuous**	385 (81.2%)	0.812 (0.752–0.877)	<0.001^*^	0.767 (0.700–0.841)	<0.001^*^	0.756 (0.678–0.843)	<0.001^*^
**Natural log transformed**	385 (81.2%)	0.083 (0.031–0.220)	<0.001^*^	0.042 (0.013–0.137)	<0.001^*^	0.040 (0.010–0.165)	<0.001^*^
**Quartile**							
**Q1 (1.63 ≤ 8.70)**	113 (95.8)	1.0 (Ref)		1.0 (Ref)		1.0 (Ref)	
Q2 (8.70 ≤ 11.40)	100 (84.0)	0.233 (0.084–0.647)	0.005 ^*^	0.059 (0.013–0.262)	<0.001^*^	0.059 (0.012–0.291)	0.001^*^
Q3 (11.40 ≤ 13.30)	95 (79.8)	0.175 (0.064–0.477)	0.001^*^	0.053 (0.012–0.238)	<0.001^*^	0.049 (0.010–0.240)	<0.001^*^
Q4 (1.30 ≤ 22.00)	77 (65.3)	0.083 (0.031–0.220)	<0.001^*^	0.024 (0.005–0.106)	<0.001^*^	0.023 (0.005–0.118)	<0.001^*^
**TIA–type MMD**							
**Continuous**	110 (55.3)	0.768 (0.688–0.857)	<0.001^*^	0.726 (0.636–0.829)	<0.001^*^	0.667 (0.559–0.796)	<0.001^*^
**Natural log transformed**	110 (55.3)	0.059 (0.016–0.222)	<0.001^*^	0.035 (0.007–0.179)	<0.001^*^	0.020 (0.002–0.164)	<0.001^*^
**Quartile**							
Q1 (1.63 ≤ 9.75)	41 (82.0)	1.0 (Ref)		1.0 (Ref)		1.0 (Ref)	
Q2 (9.74 ≤ 11.70)	28 (56.0)	0.279 (0.112 −0.696)	0.006^*^	0.247 (0.091–0.670)	0.006 ^*^	0.196 (0.055–0.697)	0.012^*^
Q3 (11.70 ≤ 13.40)	24 (48.0)	0.203 (0.082–0.503)	0.001^*^	0.189 (0.068–0.520)	0.001^*^	0.128 (0.036–0.448)	0.001^*^
Q4 (13.40 ≤ 18.70)	17 (34.7)	0.117 (0.046–0.296)	<0.001^*^	0.078 (0.026–0.237)	<0.001^*^	0.052 (0.012–0.222)	<0.001^*^
**Infarction–type MMD**							
**Continuous**	157 (63.8)	0.820 (0.750–0.897)	<0.001^*^	0.739 (0.655–0.833)	<0.001^*^	0.724 (0.615–0.852)	<0.001^*^
**Natural log transformed**	157 (63.8)	0.089 (0.030–0.264)	<0.001^*^	0.022 (0.005–0.107)	<0.001^*^	0.033 (0.005–0.226)	0.001^*^
**Quartile**							
Q1 (2.14 ≤ 9.25)	58 (95.1)	1.0 (Ref)		1.0 (Ref)		1.0 (Ref)	
Q2 (9.25 ≤ 11.70)	35 (56.5)	0.067 (0.019 −0.237)	<0.001^*^	0.029 (0.007 −0.123)	<0.001^*^	0.016 (0.002 −0.105)	<0.001^*^
Q3 (11.70 ≤ 13.80)	31 (50.0)	0.052 (0.015–0.183)	<0.001^*^	0.022 (0.005–0.096)	<0.001^*^	0.008 (0.001–0.064)	<0.001^*^
Q4 (13.80 ≤ 22.00)	33 (54.1)	0.061 (0.017–0.216)	<0.001^*^	0.021 (0.004–0.095)	<0.001^*^	0.021 (0.003–0.150)	<0.001^*^
**Hemorrhagic–type MMD moyamoya**							
**Continuous**	118 (57.0)	0.765 (0.687–0.851)	<0.001^*^	0.737 (0.651–0.835)	<0.001^*^	0.712 (0.610–0.832)	<0.001^*^
**Natural log transformed**	118 (57.0)	0.047 (0.013–0.171)	<0.001^*^	0.030 (0.006–0.138)	<0.001^*^	0.021 (0.003–0.147)	<0.001^*^
**Quartile**							
Q1 (1.85 ≤ 9.29)	48 (92.3)	1.0 (Ref)		1.0 (Ref)		1.0 (Ref)	
Q2 (9.29 ≤ 11.70)	26 (50.0)	0.083 (0.026–0.265)	<0.001^*^	0.080 (0.024 −0.263)	<0.001^*^	0.052 (0.012 −0.221)	<0.001^*^
Q3 (11.70 ≤ 13.50)	24 (46.2)	0.071 (0.022–0.227)	<0.001^*^	0.071 (0.021–0.235)	<0.001^*^	0.049 (0.011–0.214)	<0.001^*^
Q4 (13.50 ≤ 19.40)	20 (39.2)	0.054 (0.017–0.172)	<0.001^*^	0.040 (0.011–0.140)	<0.001^*^	0.023 (0.005–0.114)	<0.001^*^

*Crude model: unadjusted logistic regression model; Model 1: adjusted for age, sex, heart rate, SBP, DBP, and BMI; Model 2: further adjusted for WBC count, lymphocyte count, neutrophil count, platelet count, albumin, creatinine uric acid, triglyceride, total cholesterol, HDL cholesterol, LDL cholesterol, ApoA, ApoB, homocysteine, and eGFR*.

* *p < 0.05*.

**Table 4:** The association between baseline betaine levels and the risk of moyamoya and its subtypes.

**Betaine**, μ**mol/L**	**No. of events (%)**	**Crude model**		**Model 1**		**Model 2**	
		**OR (95%CI)**	***p***	**OR (95%CI)**	***p***	**OR (95%CI)**	***p***
**MMD overall**							
**Continuous**	385 (81.2%)	0.955 (0.940–0.971)	<0.001^*^	0.949 (0.932–0.966)	<0.001^*^	0.952 (0.932–0.972)	<0.001
**Natural log transformed**	385 (81.2%)	0.148 (0.075–0.292)	<0.001^*^	0.114 (0.053–0.245)	<0.001	0.122 (0.053–0.280)	<0.001
**Quartile**							
Q1 (4.25 ≤ 25.48)	116 (98.3)	1.0 (Ref)		1.0 (Ref)		1.0 (Ref)	
Q2 (25.48 ≤ 33.70)	95 (79.8)	0.233 (0.084–0.647)	0.005^*^	0.192 (0.067–0.549)	0.002^*^	0.213 (0.066–0.689)	0.010^*^
Q3 (33.70 ≤ 42.30)	93 (78.2)	0.175 (0.064–0.477)	0.001^*^	0.136 (0.048–0.386)	<0.001^*^	0.128 (0.040–0.411)	0.001^*^
Q4 (42.30 ≤ 358.00)	81 (68.6)	0.083 (0.031–0.220)	<0.001^*^	0.061 (0.022–0.171)	<0.001^*^	0.058 (0.018–0.184)	<0.001^*^
**TIA–type MMD**							
**Continuous**	110 (55.3)	0.951 (0.929–0.973)	<0.001^*^	0.944 (0.920–0.969)	<0.001^*^	0.940 (0.908–0.973)	<0.001^*^
**Natural log transformed**	110 (55.3)	0.154 (0.067–0.355)	<0.001^*^	0.113 (0.043–0.298)	<0.001^*^	0.090 (0.028–0.286)	<0.001^*^
**Quartile**							
Q1 (4.25 ≤ 27.80)	40 (80.0)	1.0 (Ref)		1.0 (Ref)		1.0 (Ref)	
Q2 (27.80 ≤ 35.90)	29 (58.0)	0.345 (0.141–0.842)	0.019^*^	0.234 (0.086–0.634)	0.004^*^	0.137 (0.039–0.484)	0.002^*^
Q3 (35.90 ≤ 44.00)	24 (48.0)	0.231 (0.095–0.561)	0.001^*^	0.179 (0.066–0.487)	0.001^*^	0.166 (0.049–0.560)	0.004^*^
Q4 (44.00 ≤ 358.00)	17 (34.7)	0.133 (0.054–0.330)	<0.001^*^	0.102 (0.036–0.290)	<0.001^*^	0.064 (0.017–0.246)	<0.001^*^
**Infarction–type MMD**							
**Continuous**	110 (55.3)	0.952 (0.932–0.972)	<0.001^*^	0.942 (0.919–0.966)	<0.001^*^	0.931 (0.896–0.967)	<0.001^*^
**Natural log transformed**	157 (63.8)	0.152 (0.069–0.335)	<0.001^*^	0.104 (0.041–0.267)	<0.001^*^	0.076 (0.020–0.292)	<0.001^*^
**Quartile**							
Q1 (5.31 ≤ 27.50)	54 (88.5)	1.0 (Ref)		1.0 (Ref)		1.0 (Ref)	
Q2 (27.50 ≤ 36.35)	38 (61.3)	0.205 (0.080–0.525)	0.001^*^	0.143 (0.050–0.412)	<0.001^*^	0.307 (0.080–1.185)	0.087
Q3 (36.35 ≤ 44.13)	35 (56.5)	0.168 (0.066–0.428)	<0.001^*^	0.117 (0.041–0.337)	<0.001^*^	0.104 (0.026–0.415)	0.001^*^
Q4 (44.13 ≤ 358.00)	30 (49.3)	0.125 (0.049–0.319)	<0.001^*^	0.090 (0.030–0.270)	<0.001^*^	0.106 (0.025–0.449)	0.002^*^
**Hemorrhagic–type MMD**							
**Continuous**	118 (57.0)	0.947 (0.925–0.969)	<0.001^*^	0.942 (0.919–0.966)	<0.001^*^	0.946 (0.919–0.975)	<0.001^*^
**Natural log transformed**	118 (57.0)	0.114 (0.047–0.274)	<0.001^*^	0.096 (0.037–0.248)	<0.001^*^	0.106 (0.035–0.322)	<0.001^*^
**Quartile**							
Q1 (5.10 ≤ 27.20)	45 (86.5)	1.0 (Ref)		1.0 (Ref)		1.0 (Ref)	
Q2 (27.20 ≤ 34.70)	32 (61.5)	0.249 (0.094–0.658)	0.005^*^	0.272 (0.099–0.742)	0.011^*^	0.428 (0.132–1.388)	0.158
Q3 (34.70 ≤ 43.80)	23 (44.2)	0.123 (0.047–0.324)	<0.001^*^	0.121 (0.045–0.328)	<0.001^*^	0.113 (0.035–0.370)	<0.001^*^
Q4 (43.80 ≤ 358.00)	18 (35.3)	0.085 (0.032–0.226)	<0.001^*^	0.080 (0.028–0.226)	<0.001^*^	0.084 (0.025–0.282)	<0.001^*^

*Crude model: unadjusted logistic regression model; Model 1: adjusted for age, sex, heart rate, SBP, DBP, and BMI; Model 2: further adjusted for WBC count, lymphocyte count, neutrophil count, platelet count, albumin, creatinine uric acid, triglyceride, total cholesterol, HDL cholesterol, LDL cholesterol, ApoA, ApoB, homocysteine, and eGFR*.

* *p < 0.05*.

### Choline pathway metabolites and risk of MMD subtypes

Consistently, the risk of TIA-type MMD was decreased with each increment in choline level (per 1 μmol increase: OR, 0.667; 95% CI, 0.559–0.796) and betaine level (per 1 μmol increase: OR, 0.940; 95% CI, 0.908–0.973), respectively. When choline and betaine were assessed as quartiles, compared with the lowest quartile of serum choline and betaine levels, those in the highest quartile had a significantly decreased risk of TIA-type MMD (choline, Q4 vs. Q1: OR, 0.052; 95% CI, 0.012–0.222); betaine, Q4 vs. Q1: OR, 0.064; 95% CI, 0.017–0.246).

The risk of infarction-type MMD was decreased with each increment in choline level (per 1 μmol increase: OR, 0.756; 95% CI, 0.678–0.843) and betaine level (per 1 μmol increase: OR, 0.952; 95% CI, 0.932–0.972), respectively. When choline and betaine were assessed as quartiles, compared with the lowest quartile of serum choline and betaine levels, those in the highest quartile had a significantly decreased risk of MMD (choline, Q4 vs. Q1: OR, 0.021; 95% CI, 0.003–0.150; betaine, Q4 vs. Q1: OR, 0.106; 95% CI, 0.025–0.449).

The risk of hemorrhagic-type MMD was decreased with each increment in choline level (per 1 μmol increase: OR, 0.712; 95% CI, 0.610–0.832) and betaine level (per 1 μmol increase: OR, 0.946; 95% CI, 0.919–0.975), respectively. When choline and betaine were assessed as quartiles, compared with the lowest quartile of serum choline and betaine levels, those in the highest quartile had a significantly decreased risk of MMD (choline, Q4 vs. Q1: OR, 0.023; 95% CI, 0.005–0.114; betaine, Q4 vs. Q1: OR, 0.084; 95% CI, 0.025–0.282).

## Discussion

In this case-control study, we found that serum choline and betaine levels were inversely associated with the risk of MMD. After stratified by MMD subtypes, consistently, choline and betaine levels were also related to the risk of TIA-type, infarction-type, and hemorrhagic-type MMD. Therefore, our findings suggest that metabolites from choline pathways may play a crucial role in the risk of MMD and its subtypes.

Choline is a dietary component essential for normal functions of cell membranes, cholinergic neurotransmission, transmembrane signaling, lipid transport, and one-carbon metabolism (13, 16). Dietary deficiency of choline results in fatty liver disease, hemorrhagic kidney necrosis, muscle damage, and organ dysfunction (17). In the present study, we found that serum choline levels were inversely associated with the risk of MMD and its subtypes. Though it is unclear how choline contributes to MMD, several possible explanations can be proposed. First, existing evidence indicates that low dietary intake of choline and betaine may change epigenetic patterns and regulate gene expression *via* epigenetic modifications (18). One recent study reveals that DNA methylation was involved in the pathogenesis of MMD (19), the other study suggested that DNA methylation status at the sortilin 1 promoter CpG site may be a potential biomarker for MMD (20). Second, choline and betaine are important sources of 1-carbon units (11, 13, 16), when choline stores are inadequate, methylation of Hcy to methionine is decreased resulting in increased Hcy, and increased Hcy has been related to greater risk of MMD (9). Additionally, choline deficiency increases the activity of acetylcholinesterase (21), and serum cholinesterase activities reflect the intensity of the neuroinflammatory response in patients with stroke (22). Moreover, choline and betaine are metabolites of the choline pathway associated with decreased cardiovascular risks and recurrent strokes (23, 24).

Betaine is an oxidation product of choline, which is linked to folate and serves as a methyl donor in one-carbon metabolism (12, 14, 25). A low concentration of betaine is associated with a number of stroke risk factors, such as insulin resistance, diabetes mellitus, lipid metabolism, and atherogenic dyslipidemia (24, 26–28). In the current study, we observed an inverse relationship between betaine and risk of MMD and its subtypes. For these inverse associations, several possible pathophysiologic pathways have been proposed. First, betaine converts Hcy to methionine and dimethylglycine, which may reduce Hcy-induced oxidative stress, inflammation, apoptosis, autophagy, and endothelial dysfunction (14, 29–31). Second, betaine inhibits nuclear factor-κB activity, and nod-like receptor protein 3 (NLRP3) inflammasome activation has anti-inflammatory effects (14). Additionally, experimental studies have shown that betaine could reverse platelet aggregation *in vivo* and *in vitro* and protect against coagulation events (32).

The optimal treatment for MMD still remains controversial (33, 34). Currently, revascularization surgery is a routine treatment for MMD, but surgical bypass will not reverse the MMD process, and the major goal of the revascularization surgery is to reduce the risk of recurrent stroke and improve neurological functions (34). Meanwhile, the appropriate medical treatment, before or after surgical revascularization, is almost completely ignored by scientific reports. In the present study, choline and betaine levels were also related to the risk of MMD and its subtypes, which may suggest choline and betaine supplementation might be a novel medical strategy in patients with MMD. In addition, previous studies indicated that one-carbon metabolism supplementation has some benefits on stroke outcome, which may increase neuroplasticity and recovery after stroke (14, 35, 36). Recent research finds that methylenetetrahydrofolate reductase gene (MTHFR) and transcobalamin II (TCN2), which regulate Hcy metabolism, were novel susceptibility genes for MMD (37). In addition, betaine plays a major role in determining total Hcy levels, particularly in case of folate deficiency and MTHFR mutations (38). Furthermore, an observational study found that the high intake of choline and betaine was inversely associated with inflammation (39). These results highlight the need for prospective studies that choline and betaine supplementation in patients with MMD. As a further note, it has been found that choline and betaine intakes were not related to cardiovascular disease mortality risk (40, 41), which indicates choline and betaine supplementation in patients with MMD should be taken with caution and much further investigation. There were several limitations of this study. First, all of the patients in the present study were selected from one single neurosurgery center with heterogeneous study populations and risk of bias. Second, this study included only Chinese adults with MMD, so the results cannot be generalized to pediatrics or other ethnic groups. Third, data on dietary intakes of choline and betaine, phosphocholine, sphingomyelin, or other precursors were not collected. Therefore, further investigation of the association between choline and betaine intakes and MMD was limited. Fourth, as a result of the study design, we were unable to demonstrate that choline pathway biomarkers are causally linked with MMD and its subtypes, even after considering many potential confounders. To understand the causality of these risk factors, treatment and long-term follow-up are required.

## Conclusions

Serum choline and betaine were associated with decreased risk of MMD and its subtypes.

## Data availability statement

The raw data supporting the conclusions of this article will be made available by the authors, without undue reservation.

## Ethics statement

The studies involving human participants were reviewed and approved by the Ethics Committee of Beijing Tiantan Hospital, Capital Medical University. The patients/participants provided their written informed consent to participate in this study.

## Author contributions

PG, DZ, and JZ: conception and design. YZhao, YZhai, and JW: acquisition of data. PG and YZhai: analysis and interpretation of data. PG: drafting the article. QZ, XY, RW, YZhan, and DZ: technical supports. JZ: study supervision. All authors critically revising the article and approved the final version of the manuscript.

## Funding

This study was supported by the National Key Research and Development Program of China (2021YFC2500502), Beijing Municipal Organization Department Talents Project (2015000021469G219), the National Natural Science Foundation of China (81701137), and Beijing Municipal Administration of Hospitals' Mission Plan (SML20150501).

## Conflict of interest

The authors declare that the research was conducted in the absence of any commercial or financial relationships that could be construed as a potential conflict of interest.

## Publisher's note

All claims expressed in this article are solely those of the authors and do not necessarily represent those of their affiliated organizations, or those of the publisher, the editors and the reviewers. Any product that may be evaluated in this article, or claim that may be made by its manufacturer, is not guaranteed or endorsed by the publisher.
